# An immortalized cell line derived from renal erythropoietin-producing (REP) cells demonstrates their potential to transform into myofibroblasts

**DOI:** 10.1038/s41598-019-47766-5

**Published:** 2019-08-02

**Authors:** Koji Sato, Ikuo Hirano, Hiroki Sekine, Kenichiro Miyauchi, Taku Nakai, Koichiro Kato, Sadayoshi Ito, Masayuki Yamamoto, Norio Suzuki

**Affiliations:** 1 Division of Oxygen Biology, Tohoku University Graduate School of Medicine, Sendai, Japan; 2 Division of Nephrology, Endocrinology, and Vascular Medicine, Tohoku University Graduate School of Medicine, Sendai, Japan; 3 Department of Molecular Hematology, Tohoku University Graduate School of Medicine, Sendai, Japan; 4 Department of Gene Expression Regulation, Institute of Development, Aging and Cancer, Tohoku University, Sendai, Japan; 5 Tohoku Medical Megabank Organization, Tohoku University, Sendai, Japan

**Keywords:** Stress signalling, Mechanisms of disease, Molecular medicine, Renal fibrosis

## Abstract

The erythroid growth factor erythropoietin (Epo) is produced by renal interstitial fibroblasts, called REP (renal Epo-producing) cells, in a hypoxia-inducible manner. In chronic kidney disease (CKD), REP cells lose their Epo-production ability, leading to renal anaemia. Concurrently, REP cells are suggested to be transformed into myofibroblasts, which are the major player of renal fibrosis. Although establishment of cultured cell lines derived from REP cells has been a long-term challenge, we here successfully established a REP-cell-derived immortalized and cultivable cell line (Replic cells) by using a genetically modified mouse line. Replic cells exhibited myofibroblastic phenotypes and lost their Epo-production ability, reflecting the situation in renal fibrosis. Additionally, we found that cell-autonomous TGFβ signalling contributes to maintenance of the myofibroblastic features of Replic cells. Furthermore, the promoters of genes for Epo and HIF2α, a major activator of *Epo* gene expression, were highly methylated in Replic cells. Thus, these results strongly support our contention that REP cells are the origin of myofibroblasts in fibrotic kidneys and demonstrate that cell-autonomous TGFβ signalling and epigenetic silencing are involved in renal fibrosis and renal anaemia, respectively, in CKD. The Replic cell line is a useful tool to further investigate the molecular mechanisms underlying renal fibrosis.

## Introduction

Because kidneys are the major organ producing the erythroid growth factor erythropoietin (Epo), Epo-deficiency anaemia often arises as a complication of chronic kidney disease (CKD)^1^. In addition to renal anaemia, renal fibrosis, in which myofibroblasts producing extracellular matrix emerge in peritubular interstitia, is commonly observed among a large number of CKD patients^2^. Renal myofibroblasts are considered to originate in diverse ways, for example, through transformation of tubular epithelial cells^3^, capillary endothelial cells^4^, and interstitial fibroblasts^5–7^ in kidneys as well as the migration of bone marrow cells^8,9^. Of the multiple myofibroblast origins, interstitial fibroblasts are thought to mainly contribute to renal fibrosis in response to inflammation signalling activated by injury to nephrons^6–9^. Importantly, interstitial fibroblasts distributed in renal cortices and corticomedullary boundaries are the site of Epo production^10–13^, and the Epo-production ability of fibroblasts (renal Epo-producing [REP] cells) is inactivated by myofibroblastic transformation^6,7^. Thus, renal anaemia and fibrosis are tightly related to each other in REP cells during CKD progression.

REP cells express the *Epo* gene in a hypoxia-inducible manner to maintain a systemic oxygen supply *via* erythrocytes^14^. For hypoxic induction of the *Epo* gene, hypoxia-inducible transcription factor 2α (HIF2α) is essential^15^. The α subunits of hypoxia-inducible factors (HIFs), including HIF2α, are degraded and inactivated under normal oxygen conditions (normoxia) through hydroxylation of their specific proline residues followed by ubiquitin-proteasome degradation, whereas hypoxia stabilizes and activates HIFs by blocking hydroxylation^16–18^. Prolyl hydroxylase domain enzymes (PHDs) are hypoxia-sensing molecules that catalyse the hydroxylation of HIFs in an oxygen-dependent manner^19^. We previously reported that HIF2α is inactivated in myofibroblast-transformed REP (MF-REP) cells in damaged mouse kidneys regardless of their hypoxic milieu^15^. Because forced activation of HIF2α by deletion of genes for PHDs restores the Epo-production ability in MF-REP cells, inactivation of HIF2α in MF-REP cells is considered to be due to abnormal activation of PHDs in hypoxic myofibroblasts^15^. HIF2α is one of the most important molecules in renal anaemia development. In fact, chemicals that are inhibitory to PHDs (PHDi) are undergoing clinical trials for renal anaemia treatment^20^.

The myofibroblastic transformation of REP cells is reversed after the restoration of kidney injuries at least partially, and inflammatory signalling such as transforming growth factor β (TGFβ) and tumour necrosis factor α signalling, are involved in this transformation^7^. Although understanding the mechanisms of renal fibrosis is critical for elucidating renal anaemia and CKD progression, the molecular characterization of REP cells has not been investigated due to the lack of appropriate culture cell models for REP cells. In this study, we isolated REP cells from mouse kidneys and immortalized them by the exogenous expression of oncogenic H-RAS. Consequently, one cell line referred to as “Replic (REP-cell lineage cells immortalized and cultivable) cells” was successfully established. Replic cells exhibited myofibroblastic features with high-level TGFβ expression, and inhibition of TGFβ signalling attenuated the myofibroblast-related gene expression pattern in the cells. Additionally, the cells lost their Epo-production ability by epigenetic suppression of HIF2α expression. These results directly indicate that renal myofibroblasts emerge from transformation of REP cells in injured kidneys and that the cell-autonomous TGFβ signal is involved in REP cell transformation to myofibroblasts. Furthermore, it is demonstrated that epigenetic silencing of genes for Epo and/or HIF2α is one of the major causes for loss of the Epo-production ability in MF-REP cells. We also propose that Replic cells are a valuable tool to understand the mechanisms underlying renal fibrosis and renal anaemia, both of which are significant complications of CKD associated with disease progression^21,22^.

## Results

### Cultivation and immortalization of REP cells isolated from ISAM-REC mice

We previously established a gene-modified mouse line in which REP cells are efficiently labelled with tdTomato red fluorescent protein expression^13^. In this mouse line, referred to as ISAM-REC mice (*Epo*^*GFP/GFP*^*:Rosa26*^*CAG-LSL-tdTomato/WT*^*:Tg*^*3*.*3K-Epo*^*:Tg*^*EpoCre*^ genotype)^14^, the expression of transgenic Cre recombinase under the control of the *Epo* gene regulatory region is highly induced by severe anaemic conditions due to Epo deficiency, and most REP cells permanently express tdTomato as a marker for Cre-mediated recombination without any treatment^14,23^. Thus, tdTomato-positive cells from ISAM-REC kidneys were applied for *ex vivo* cultivation and immortalization to generate cell lines derived from REP cells.

**Figure 1:**
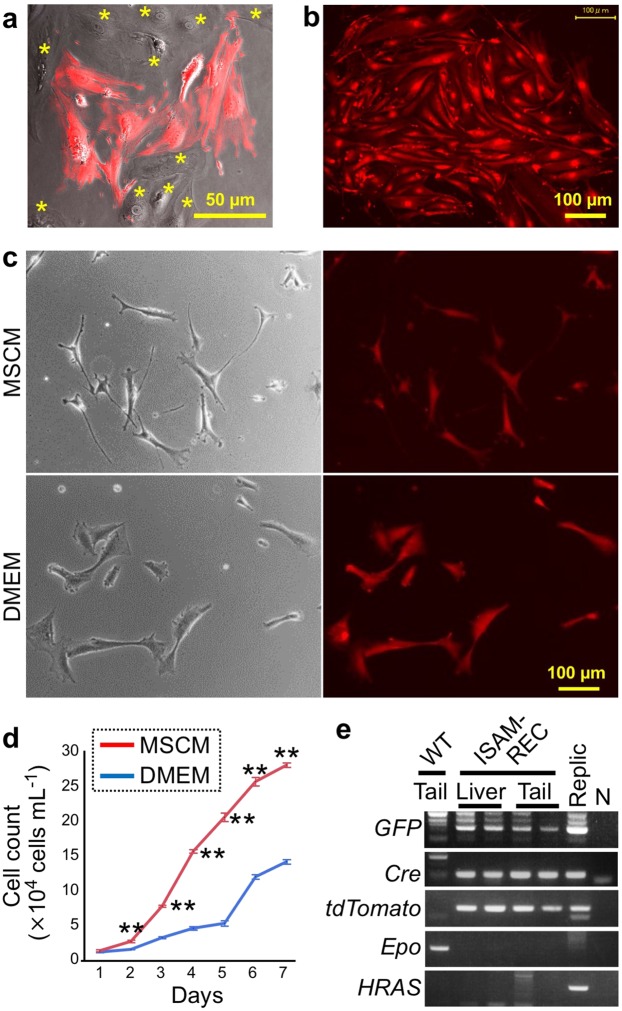
Establishment of a Replic cell line. (**a**) A representative image of cells cultured in MSCM for one week after isolation from ISAM-REC kidneys. The mixed cell culture contains cells positive (red) and negative (asterisk) for tdTomato fluorescence. (**b**) A colony of tdTomato-positive cells was generated after immortalization and isolation of tdTomato-positive REP cell lineage cells from the mixed renal cell culture. (**c**) Phase-contrast (left) and tdTomato fluorescent (right) images of Replic cells cultured with MSCM (upper) or DMEM (lower). (**d**) Growth curves of Replic cells cultured with DMEM (blue) or MSCM (red). On Day 0, 1.0 × 10^4^ cells were seeded onto 3.5-cm dishes with 2.5 mL medium, and cell numbers were counted daily. The data are shown as the means ± standard errors. n = 3 for each group. **p < 0.01 by two-tailed and unpaired Student’s t tests. (**e**) Genomic PCR of Replic cells compared with that of organs from wild-type C57BL/6 (WT) and ISAM-REC mice. N, a non-template negative control.

First, tdTomato-positive cells were isolated from ISAM-REC kidney cell suspensions by using a cell sorter, and the cells were incubated with mesenchymal stem cell growth medium (MSCM). However, no cells survived after the 10-day *ex vivo* cultivation, suggesting that cell-cell communications and/or soluble factors secreted by kidney cells other than REP cells are required for REP cell expansion and maintenance. Therefore, the cell suspensions were directly incubated without cell sorting. After a week of cultivation, we observed that tdTomato-positive cells grew with tdTomato-negative cells, attaching to the bottoms of culture dishes (Fig. 1a).

Because it was considered that the 1-week cultivation let the kidney cells adapt to the culture conditions, the cells were integrated with a lentivirus vector expressing the human *HRAS* gene bearing an oncogenic mutation in order to immortalize the cells. After expansion of the infected cells for 2 weeks, tdTomato-positive REP cells in the dishes were sorted and subjected to pure cultivation. Finally, a single colony of tdTomato-positive cells was developed (Fig. 1b). It was possible for the colony-derived cells in culture dishes to grow with MSCM for more than 1 year, to recover from freeze-thawing, and to be passaged more than 10 times. We thus concluded that a REP-cell-derived cell line was established and named the cells Replic (REP cell-lineage immortalized and cultivable) cells.

### Growth of Replic cells

Replic cells and natural REP cells in ISAM-REC mice shared a fibroblast-like shape that exhibited bipolar stretching with bright fluorescence of tdTomato (Fig. 1c)^12^. The cell shape was narrow and stretched with many processes when the cells were cultured with MSCM rather than with Dulbecco’s modified Eagle’s medium (DMEM) supplemented with 10% foetal bovine serum (FBS) (Fig. 1c). This observation suggests that MSCM is a more appropriate culture medium than DMEM for maintaining the features of *in vivo* REP cells under *ex vivo* culture conditions because the REP cells show a sharp and extended morphology with many processes in the renal interstitia^11,12^. Therefore, MSCM was used for characterization of Replic cells in this study. Replic cells proliferated significantly in MSCM rather than in DMEM and doubled daily in the logarithmic growth phase on normal polystyrene dishes without any coating (Fig. 1d). Although the contents of MSCM are veiled by the manufacturer, the cell-growth-optimized MSCM likely promotes proliferation of Replic cells compared with the basic medium.

Genotyping PCR confirmed that the immortality and proliferative activity of Replic cells were supported by integration of the oncogenic mutant *HRAS* gene (Fig. 1e). The ISAM-REC kidney origin of Replic cells was confirmed by PCR data demonstrating that Replic cells and ISAM-REC mice commonly harboured the *Cre*, *tdTomato*, and *GFP* genes but lacked the *Epo* gene (Fig. 1e). From these results, we concluded that the first REP-cell-derived cell line has been established and that the cells vigorously proliferate, maintaining their fibroblastic features under culture conditions optimized for mesenchymal stem cells.

### Fibroblastic phenotype of Replic cells

**Figure 2:**
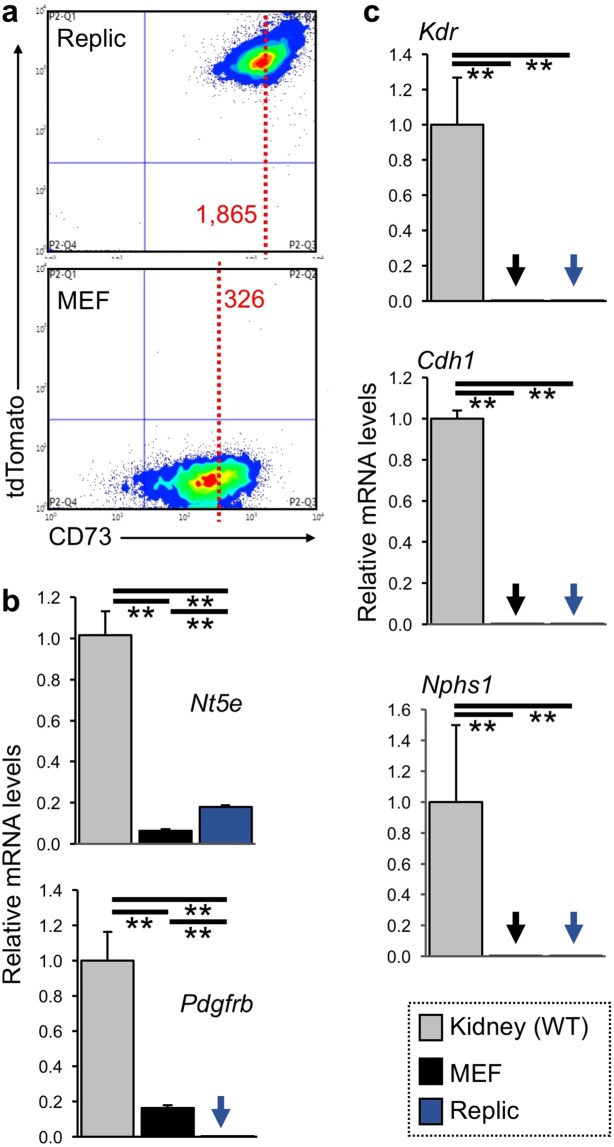
Fibroblastic phenotype of Replic cells. (**a**) Flow cytometry of Replic cells (upper) and MEFs (lower) cultured with MSCM. Every Replic cell expressed high-level tdTomato fluorescence. The mean fluorescent intensity of CD73 in Replic cells was higher than that in MEFs (dotted red lines). (**b**,**c**) The mRNA expression levels of marker genes for fibroblasts (**b**) and non-fibroblastic renal cells (**c**) in Replic cells were compared to those in wild-type (WT) kidneys and MEFs. Arrows indicate undetectable levels. Cell culture was conducted with MSCM. Ten biologically independent samples for each cell line were analysed. The average expression level of WT kidneys (n = 3) was set at 1.0, and error bars indicate standard errors. **p < 0.01 by multiple comparisons using one-way ANOVA with Tukey-Kramer tests.

Flow cytometry of Replic cells detected high-level tdTomato fluorescence in every Replic cell (Fig. 2a), showing the clonality of the cell line. The data also revealed that compared to wild-type mouse embryonic fibroblasts (MEFs), Replic cells strongly expressed CD73 (ecto-5′-nucleotidase), a fibroblastic surface marker of REP cells in mouse kidneys^12,24,25^. Consistently, gene expression analyses showed that Replic cells expressed the gene for CD73 (*Nt5e*) at higher levels than MEFs did (Fig. 2b).

Replic cells did not express the *Kdr*, *Cdh1*, and *Nphs1* genes, which are lineage markers for vascular endothelial cells, tubular epithelial cells, and podocytes, respectively (Fig. 2c). Taken together, the findings revealed that the cell line originated from tdTomato^+^CD73^+^ REP cells of the ISAM-REC kidney but not from other lineage cells of kidneys. However, mRNA expression of the *Pdgfrb* gene encoding PDGFRβ (platelet-derived growth factor receptor β), another fibroblastic marker of REP cells^6,12,26^, was undetectable in Replic cells (Fig. 2b). These data suggested that the cell phenotype was altered during isolation, cultivation, and/or immortalization.

### *Epo* gene activity and hypoxic response of Replic cells

Because the *Epo* gene of ISAM-REC mice was homozygously replaced with the gene for green fluorescent protein (GFP), the *Epo* gene activity of Replic cells derived from an ISAM-REC mouse was able to be evaluated by measuring the amounts of mRNA transcribed from the *EpoGFP* recombinant gene^13,27^. In REP cells, *Epo* gene expression is induced in a hypoxia-inducible manner *via* stabilization and activation of HIF2α^15^. Therefore, we investigated *EpoGFP* mRNA levels in Replic cells incubated under hypoxic (1% O_2_) conditions.

**Figure 3:**
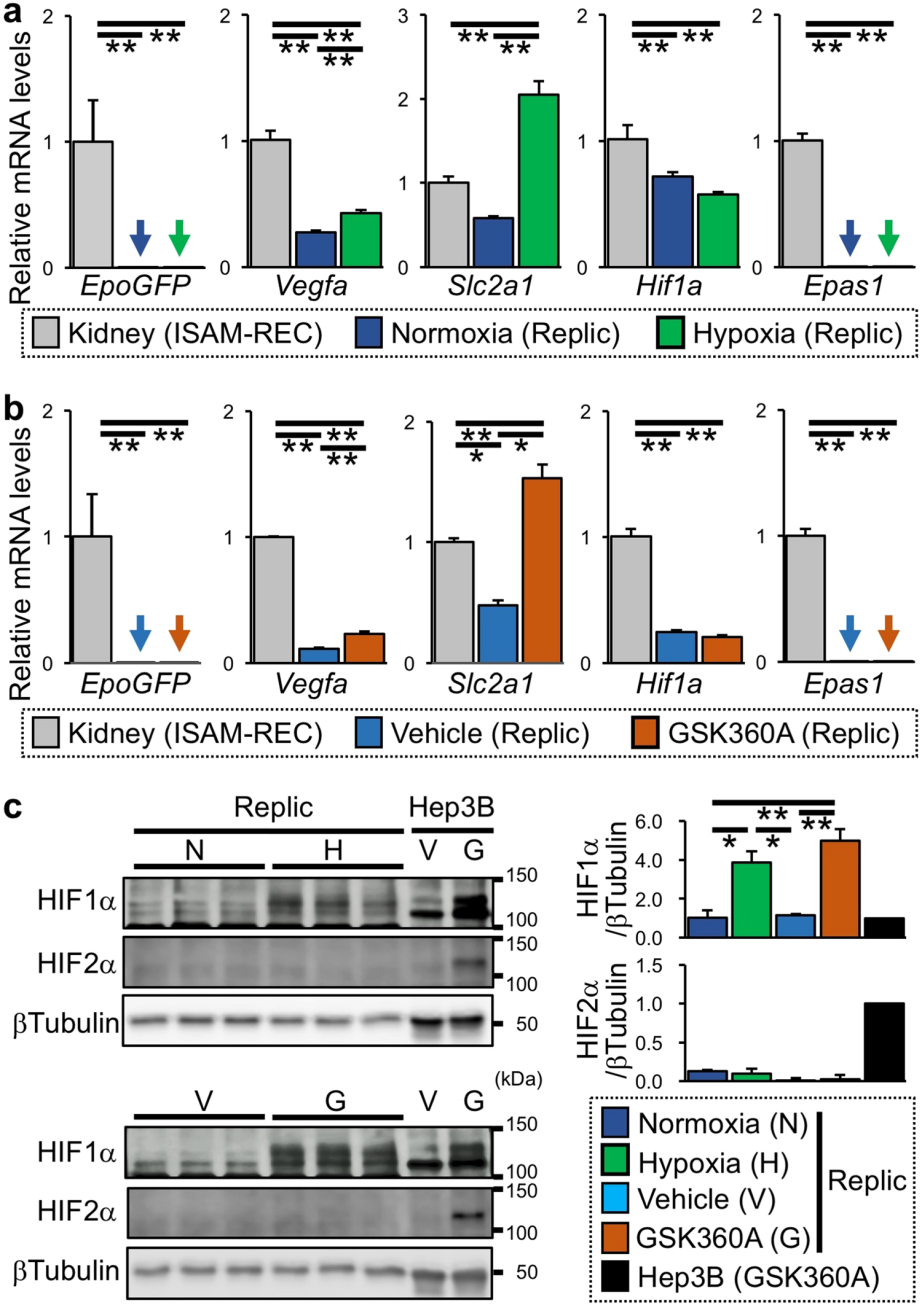
Hypoxic response of Replic cells. (**a**,**b**) mRNA expression levels of genes related to the hypoxic response in Replic cells exposed to hypoxia (**a**) or GSK360A (**b**) for 24 hours were compared to those in ISAM-REC kidneys. Twelve biologically independent samples for each group of Replic cells were analysed. The average expression level of ISAM-REC kidneys (n = 3) was set at 1.0, and error bars indicate standard errors. Arrows indicate undetectable levels. *p < 0.05 and **p < 0.01 by multiple comparisons using one-way ANOVA with Tukey-Kramer tests. (**c**) Immunoblots for HIF1α and HIF2α in Replic cells cultured under normoxic (N) or hypoxic (H, 1% oxygen) conditions for 24 hours (upper) and in Replic cells cultured with or without GSK360A (G and V, respectively) for 24 hours (lower). The HIF1α- and HIF2α-derived band intensities normalized with βTubulin-derived band intensities were quantified and are shown in the right panels. Hep3B cells was used as positive controls for the hypoxia-inducible accumulation of HIF1α and HIF2α. βTubulin was used as an internal control. Cell culture was conducted with MSCM (**a**–**c**).

In ISAM-REC kidneys, *EpoGFP* mRNA was highly expressed by severe anaemia, in agreement with our previous report (Fig. 3a,b)^27^. However, *EpoGFP* mRNA expression was undetectable in Replic cells regardless of the presence of hypoxic stimuli, while the 24-hour hypoxic exposure induced expression of the *Vegfa* and *Slc2a1* genes (encoding vascular endothelial cell growth factor A and glucose transporter 1, respectively), both of which are well known as HIF target genes (Fig. 3a)^28,29^. Additionally, expression of the gene for HIF2α (*Epas1*) was undetectable in both normoxic and hypoxic Replic cells, whereas *Hif1a* mRNA was consistently expressed under both conditions (Fig. 3a).

We then tested effects of the PHDi GSK360A^27^ on *EpoGFP* mRNA expression of Replic cells. The results showed that, similar to hypoxic exposure, GSK360A treatment induced neither *EpoGFP* nor *Epas1* expression (Fig. 3b). Because GSK360A enhanced the expression of the *Vegfa* and *Slc2a1* genes (Fig. 3b), it was demonstrated that GSK360A activated HIF signalling in Replic cells. Indeed, HIF1α protein accumulated in Replic cells after GSK360A treatment, and hypoxic exposure also induced HIF1α accumulation (Fig. 3c). Additionally, GSK360A treatment resulted in the accumulation of both HIF1α and HIF2α in a Hep3B human hepatoma cell line (Fig. 3c). However, in accordance with the data showing the absence of *Epas1* mRNA in Replic cells, HIF2α protein was undetectable in the cells even in the presence of GSK 360A or a hypoxic stimulus (Fig. 3c).

### DNA methylation in the promoters of genes for Epo and HIF2α

We surmised that epigenetic silencing blunted *Epo* and *Epas1* gene expression in Replic cells. It has been reported that DNA methylation in the *Epo* gene promoter region is involved in the loss of Epo production in fibrotic kidneys^30^. Bisulfite genomic sequence analyses of the *Epo* and *Epas1* gene promoters in Replic cells were conducted by a comparison with ISAM-REC livers, in which the *Epo* gene promoter is active in the majority of cells^13^.

**Figure 4:**
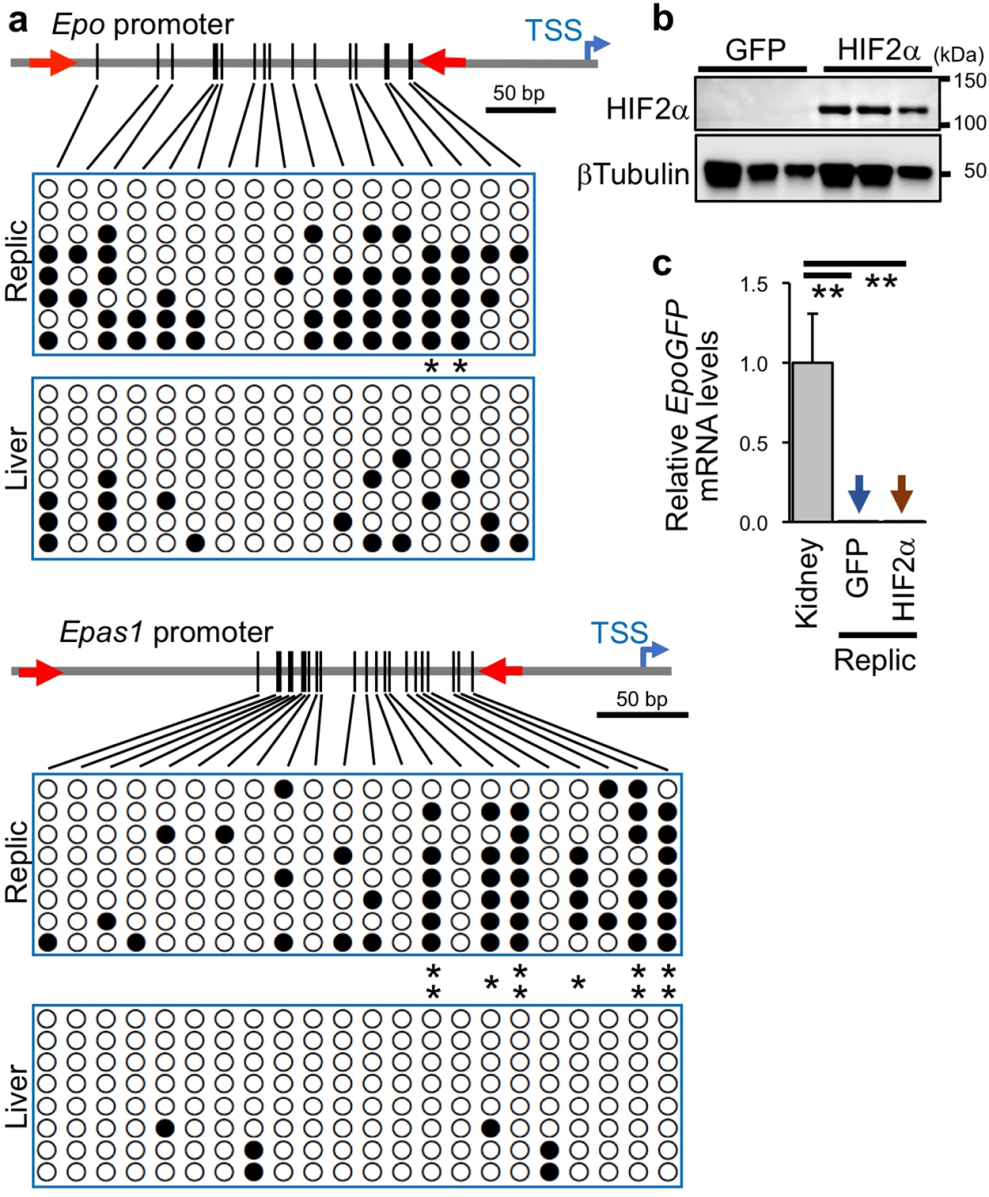
Epigenetic suppression of the Epo-production ability in Replic cells. (**a**) Summary of results from bisulfite sequencing in the *Epo* (upper) and *Epas1* (lower) gene promoters of Replic cells and ISAM-REC livers. Genomic regions between red arrows were sequenced after cloning 8 clones for each promoter from bisulfite-converted genomic DNAs. Each row represents a single clone. Vertical bars indicate CpG sites in the tested regions, and white and black dots represent unmethylated and methylated CpG sites, respectively. TSS, transcription start site. *p < 0.05 and **p < 0.01 between the methylated ratio of each CpG site in Replic cells and ISAM-REC livers, using χ^2^ tests. (**b**) Immunoblots for HIF2α in Replic cells transiently transfected with plasmids expressing GFP or constitutively active HIF2α. βTubulin was used as an internal control for immunoblots. (**c**) *EpoGFP* mRNA expression levels in Replic cells transiently transfected with plasmids expressing GFP or constitutively active HIF2α. ISAM-REC kidneys were used as positive controls for *EpoGFP* mRNA expression. Six biologically independent samples for each group of Replic cells were analysed. The average expression level of ISAM-REC kidneys (n = 3) was set at 1.0, and error bars indicate standard errors. Arrows indicate undetectable levels. **p < 0.01 by multiple comparisons using one-way ANOVA with Tukey-Kramer tests. Cell culture was conducted with MSCM (**a**–**c**).

The results revealed that the proximal promoter regions of both genes were significantly methylated in Replic cells, whereas a few CpG methylations were detected in the promoters of the ISAM-REC livers (Fig. 4a). These data suggest that the expression of Epo and HIF2α in Replic cells was silenced by DNA methylation in their gene promoters. Additionally, it was thought that Replic cells may undergo transformation to myofibroblasts, in which the *Epo* gene is methylated and inactivated^30^, maintaining their fibroblastic features.

A 1-week incubation with 5-aza-2′-deoxycytidine (5-Aza), an inhibitor of DNA methyltransferase 1 (DNMT1), which is essential for maintenance of DNA methylation patterns beyond mitosis^31^, did not alter DNA methylation of the *Epo* gene promoter region, whereas the upstream enhancer region (the -8kHRE-flanking region) of the *Epo* gene^32^ was significantly demethylated by the inhibitor in Replic cells (Supplementary Fig. 1). Therefore, the contribution of continuous *de novo* DNA methylation to the hyper-methylation of *Epo* and *Epas1* promoters in Replic cells was assumed^33^. We then tested whether exogenous HIF2α overexpression could activate *EpoGFP* gene expression in Replic cells. Constitutively active HIF2α, in which PHD-target prolyl residues were replaced with alanine residues^34^, was transiently overexpressed in Replic cells (Fig. 4b). However, *EpoGFP* expression was not induced (Fig. 4c), indicating that exogenous HIF2α overexpression was insufficient to reactivate the Epo-production ability in Replic cells. Thus, we propose that Replic cells lose their Epo-production ability due to continuous DNA methylation in the *Epo* gene promoter, which blocks the transcriptional activation signal of HIF2α.

### Myofibroblastic property of Replic cells

**Figure 5:**
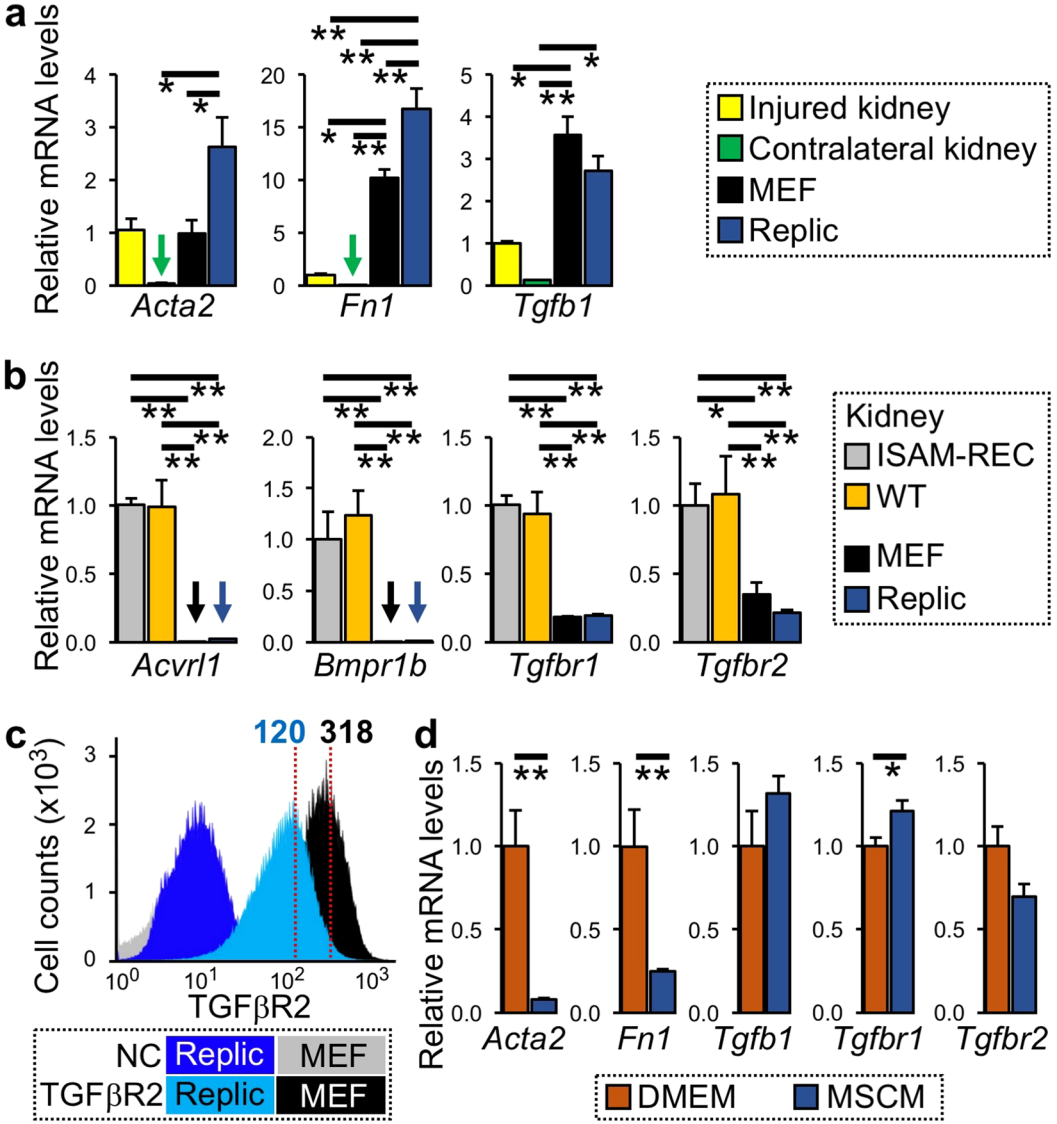
Myofibroblastic property of Replic cells. (**a**) mRNA expression levels of genes related to myofibroblasts in Replic cells cultured with MSCM. In addition to MEFs cultured with DMEM, injured and contralateral kidneys (n = 3 for each) of UUO-treated normal mice were compared to Replic cells with respect to mRNA expression. Twelve biologically independent samples for each cell line were analysed. (**b**) mRNA expression levels of genes for TGFβ superfamily receptors in Replic cells cultured with MSCM were compared to those in mouse kidneys (n = 3 for ISAM-REC kidneys and WT kidneys) and MEFs. Six biologically independent samples for each cell line were analysed. (**c**) Flow cytometry of TGFβR2 expression on the cell surface of Replic cells and MEFs cultured with DMEM. Negative controls (NC) were stained only with APC-conjugated streptavidin. The mean fluorescent intensities are shown (red dotted lines). (**d**) mRNA expression levels of genes related to myofibroblasts in Replic cells cultured with DMEM or MSCM. Six biologically independent samples for each cell line were analysed. The average expression levels of injured kidneys (**a**), ISAM-REC kidneys (**b**), or Replic cells cultured with DMEM (**d**) were set at 1.0. Error bars are indicated standard errors, and arrows indicate undetectable levels (**a**,**b**). *p < 0.05 and **p < 0.01 by multiple comparisons using one-way ANOVA with Tukey-Kramer tests (**a**,**b**) or by two-tailed, unpaired Student’s *t*-tests (**d**).

Because REP cells transform to myofibroblasts in injured kidneys with loss of the Epo-production ability and DNA hyper-methylation of the *Epo* gene promoter^6,7,30^, the myofibroblastic property of Replic cells was investigated by a comparison to fibrotic kidneys of normal mice subjected to unilateral ureteral obstruction (UUO) for 14 days. Ureteral obstruction is an established model of renal fibrosis^7,35^ and Masson’s trichrome staining of kidney sections confirmed that our ureteral obstruction surgery induced renal fibrosis (Supplementary Fig. 2a)^15^. Although MEFs were used in this experiment as the normal fibroblast control, strong expression of myofibroblastic genes was reported in MEFs^36^. Gene expression analyses of Replic cells revealed that the cells highly expressed the *Acta2* and *Fn1* genes, both of which are known to be expressed in myofibroblasts^37,38^, compared to the expression levels in MEFs or injured kidneys (Fig. 5a). The data clearly indicated that Replic cells exhibit myofibroblastic properties.

TGFβ is known to be secreted by fibrotic kidneys and to promote organ fibrosis^7,39–41^. In fact, our analyses clearly demonstrated that mRNA expression of the gene for TGFβ (*Tgfb1*) was dramatically induced by kidney injury (Fig. 5a). Notably, Replic cells expressed the *Tgfb1* gene at a comparable level to that in injured kidneys (Fig. 5a). The expression levels of genes for the major TGFβ receptors TGFβR1 (*Tgfbr1*) and TGFβR2 (*Tgfbr2*) were also detected in Replic cells, while other TGFβ superfamily receptor genes (*Acvrl1* and *Bmpr1b*) were not expressed (Fig. 5b). Flow cytometry confirmed that TGFβR2 is apparently expressed on the cell surface of the majority of Replic cells (Fig. 5c). Additionally, the gene expression levels of these receptors were significantly higher in injured kidneys than in contralateral kidneys of UUO-subjected mice (Supplementary Fig. 2b), supporting the idea that TGFβ signalling is involved in progression of renal fibrosis^42^. These data led us to assume that cell-autonomous TGFβ signalling is involved in the myofibroblastic property of Replic cells.

Based on the cell shape described above, we proposed that Replic cells seemed to differentiate into myofibroblasts by changing the culture medium from MSCM to DMEM (see Fig. 1c). This proposal was supported by the gene expression data showing that expression levels of the myofibroblastic marker genes *Acta2* and *Fn1* were significantly higher in Replic cells cultured with DMEM than in Replic cells cultured with MSCM (Fig. 5d). In contrast, the expression levels of genes for TGFβ and TGFβ receptors were not responsive to changes in the medium (Fig. 5d). Because TGFβ signalling activates expression of the *Acta2* and *Fn1* genes but not the *Tgfb1*, *Tgfbr1*, and *Tgfbr2* genes^38,43^, MSCM likely contains factors that inhibit TGFβ signalling, selectively suppressing *Acta2* and *Fn1* gene expression in Replic cells.

### Activation of cell-autonomous TGFβ signalling in Replic cells

**Figure 6:**
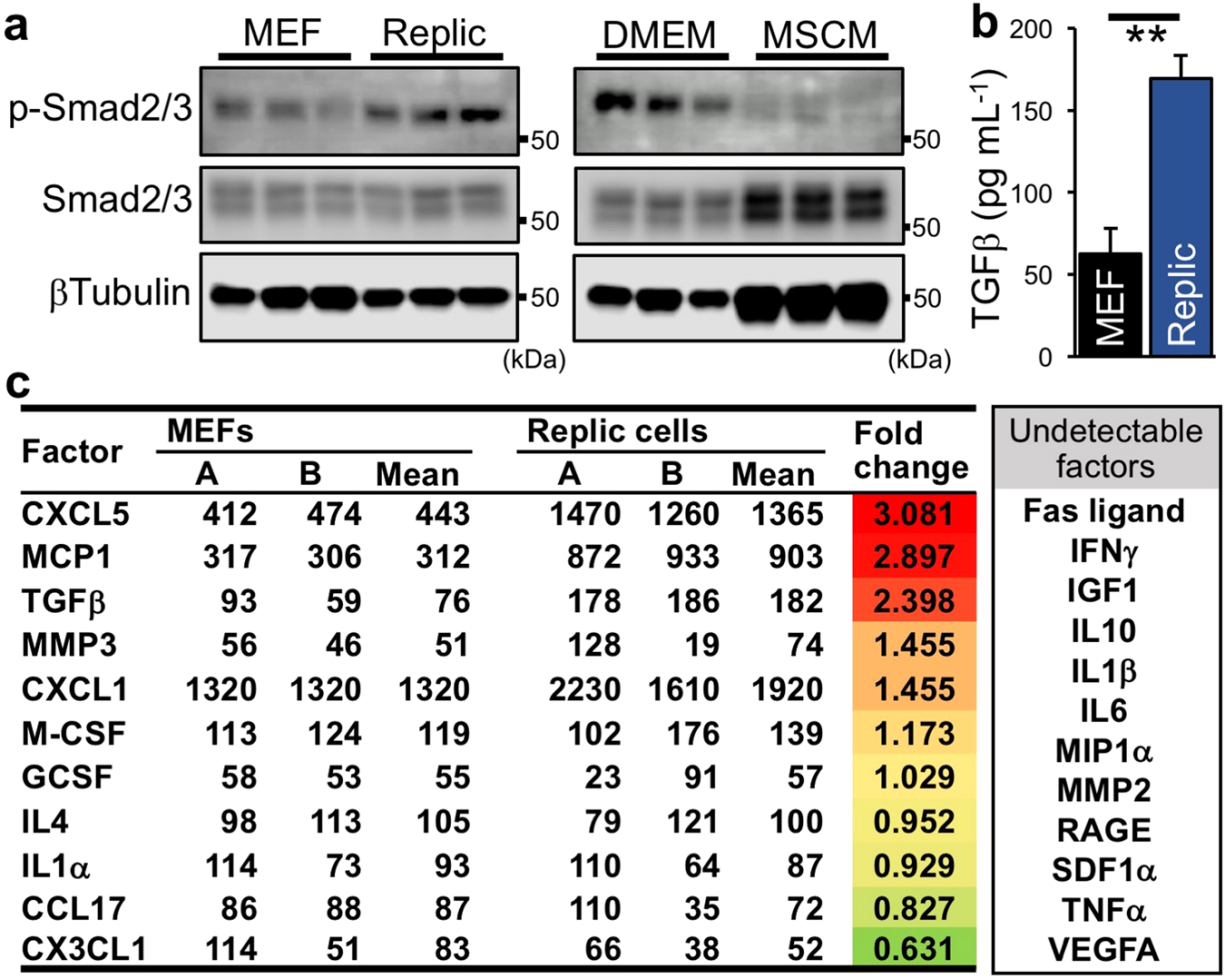
Upregulation of profibrotic TGFβ-signalling in Replic cells. (**a**) Immunoblots for p-Smad2/3 and total Smad2/3 in Replic cells cultured with DMEM were compared to those in MEFs cultured with DMEM (left) or Replic cells cultured with MSCM (right). βTubulin was used as an internal control. (**b**) TGFβ concentrations in serum-free DMEM incubated with Replic cells or MEFs for 24 hours. Three biologically independent samples for each cell line were analysed. (**c**) Relative amounts of the listed factors in culture supernatants of Replic cells and MEFs, both of which were cultured with serum-free DMEM for 24 hours, were determined by the antibody array based on a double antibody sandwich assay. The data indicate the chemiluminescent intensities of each spot (duplicate for each antibody, A and B). Undetectable cytokines, both spots of which showed signal intensities lower than 50, are listed in the right column. The scanned image of the antibody array is shown in Supplementary Fig. 3.

In the TGFβ signal, TGFβ binding to a TGFβR1/TGFβR2 heterodimeric receptor results in phosphorylation of Smad2 and/or Smad3 transcription factors followed by induction of *Acta2* and *Fn1* gene expression^44^. Consistent with the myofibroblastic gene expression profiles (Fig. 5a,d), Smad2 and/or Smad3 (Smad2/3) were highly phosphorylated in Replic cells compared to MEFs when cells were cultured with DMEM, and the phosphorylation was reduced by cultivation with MSCM (Fig. 6a). Consequently, cell-autonomous TGFβ signalling through TGFβR1/TGFβR2-Smad2/3 was considered one of the major pathways promoting myofibroblastic transformation of Replic cells.

To confirm TGFβ production by Replic cells, TGFβ concentrations in the supernatant incubated with Replic cells for 24 hours were measured by enzyme-linked immunosorbent assay (ELISA). The data demonstrated that Replic cells produce significantly higher amounts of TGFβ than MEFs (Fig. 6b), although these cell lines expressed comparable levels of *Tgfb1* mRNA (Fig. 5a). ELISA data from mouse plasma showed that plasma TGFβ concentrations are elevated in mice suffering from renal fibrosis (Supplementary Fig. 2c). These data suggest that TGFβ is secreted by fibrotic kidney cells including myofibroblast-transformed REP cells.

We further performed an antibody-based cytokine array on the culture supernatants. In agreement with the ELISA data, intense signals were detected in samples from Replic cells compared to those from MEFs (Fig. 6c, Supplementary Fig. 3a,b). The data also showed that Replic cells produced proinflammatory cytokines such as MCP1 (monocyte chemotactic protein 1, also known as CCL2) and CXCL5 (C-X-C chemokine ligand 5) at higher levels than MEFs (Fig. 6c, Supplementary Fig. 3a,b). These results suggest that Replic cells acquire myofibroblastic properties related to cell-autonomous TGFβ signalling during isolation, cultivation, and/or immortalization of fibroblastic REP cells.

### TGFβ-dependent myofibroblastic property of Replic cells

**Figure 7:**
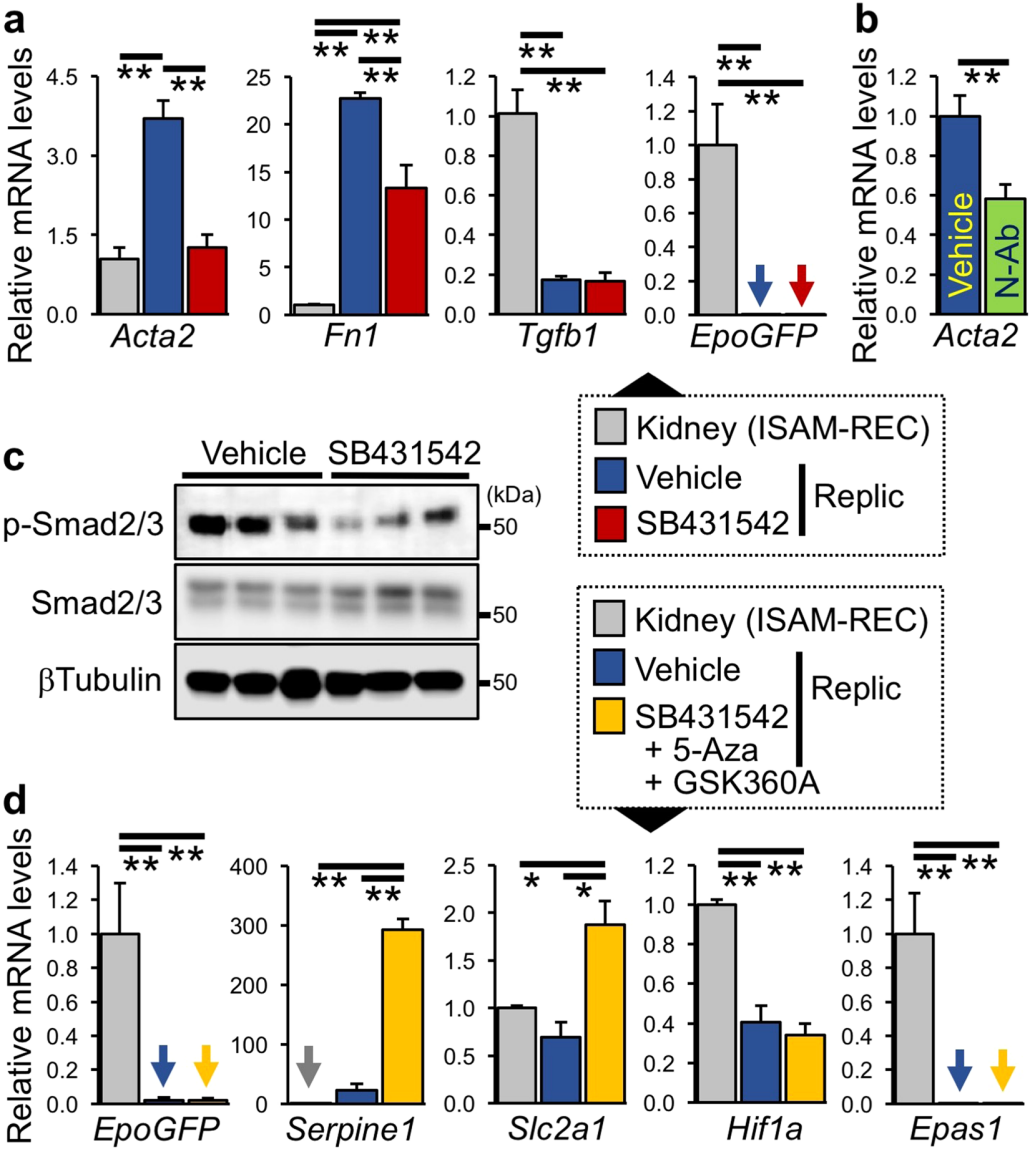
TGFβ-dependent myofibroblastic property of Replic cells. (**a**) mRNA expression levels of genes related to myofibroblasts in Replic cells incubated with or without SB431542 in DMEM for 24 hours. *EpoGFP* mRNA levels were also examined. ISAM-REC kidneys (n = 3) were used as controls. Ten biologically independent samples for each treatment of Replic cells were analysed. (**b**) The *Acta2* mRNA expression levels in Replic cells incubated with vehicle or neutralizing antibody against TGFβ (N-Ab) in DMEM for 24 hours. Six biologically independent samples for each treatment of Replic cells were analysed. (**c**) Immunoblots for p-Smad2/3 and total Smad2/3 in Replic cells cultured with or without SB431542 in DMEM for 1 hour. βTubulin was used as an internal control. (**d**) Relative mRNA expression levels of genes related to hypoxic response were measured in Replic cells cultured with DMEM in the presence or absence (Vehicle) of the inhibitor cocktail. ISAM-REC kidneys (n = 3) were used as controls. As the inhibitor cocktail, SB431542, 5-Aza, and GSK360A were added to the cell culture at 24 hours, 5 days, and 24 hours before sampling, respectively. Five biologically independent samples for each treatment of Replic cells were analysed. The average expression level of ISAM-REC kidneys (**a**,**d**) or vehicle-treated Replic cells (**b**) was set at 1.0, and the error bars represent standard errors for each experimental group. (**a**,**b**,**d**) Arrows indicate undetectable levels. *p < 0.05 and **p < 0.01 by multiple comparisons using one-way ANOVA with Tukey-Kramer tests (**a**,**d**) or by two-tailed, unpaired Student’s *t*-tests (**b**).

To investigate the necessity of cell-autonomous TGFβ signalling for the myofibroblastic property of Replic cells, Replic cells were incubated with SB431542, a kinase inhibitor specific for TGFβR1, ALK4 (activin receptor-like kinase 4), and ALK7^45^. The mRNA expression levels of the *Acta2* and *Fn1* genes were markedly decreased by SB431542, while *Tgfb1* mRNA expression was unaltered (Fig. 7a). In addition to SB431542, TGFβ neutralizing antibody (TGFβ N-Ab) reduced *Acta2* gene expression in Replic cells (Fig. 7b). Although it was predicted that the original fibroblastic property of REP cells was, at least in part, restored by inhibition of TGFβ signalling in Replic cells, *EpoGFP* mRNA expression was still undetectable in Replic cells cultured with SB431542 for 24 hours (Fig. 7a). Inhibition of TGFβ signalling by SB431542 was confirmed by immunoblotting data showing a decrease in phosphorylated Smad2/3 (p-Smad2/3, Fig. 7c).

Finally, we tested whether the Epo-production ability could be restored by simultaneous supplementation with SB431542, GSK360A, and 5-Aza. As the result, the inhibitor cocktail did not activate *EpoGFP* mRNA expression, while the expression of other HIF target genes was significantly induced (*Serpine1* and *Slc2a1*, Fig. 7d)^29,46^. The *Epas1* gene remained silent under the cocktail presence, although *Hif1a* mRNA was constantly expressed in Replic cells with or without the cocktail (Fig. 7d). Therefore, expression of the *Serpine1* and *Slc2a1* genes in Replic cells is thought to be driven through HIF1α activation by the inhibitor cocktail, which includes a PHDi. Consequently, we propose that Replic cells would be useful for elucidating the mechanisms of the myofibroblastic transformation of REP cells even though the cells may irreversibly lose their Epo-production ability.

## Discussion

Because REP cells play a central role in renal anaemia and renal fibrosis in CKD patients, cell lines derived from REP cells have long been waited for use in studies elucidating the molecular pathophysiology of REP cells. In this study, we successfully generated a REP-cell-derived cell line “Replic cells” for the first time. Because Replic cells exhibit myofibroblastic features and defects in Epo production, we are certain that Replic cells will allow us to understand the molecular mechanisms underlying the development of renal anaemia and fibrosis during kidney disease progression.

We previously reported that REP cells are inactive in proliferation and energy metabolism in healthy kidneys, whereas kidney injuries stimulate the proliferation of REP cells with Ki67 expression^7,15^. In this study, we observed that REP cells isolated from single-cell suspensions of mouse kidneys failed to survive for more than 1 week under any culture conditions tested here. Then, we tested mixed cell cultures of renal cells and found that REP cells require intercellular communication with renal cells for survival and growth *in vitro*. During the mixed culture, REP cells gained proliferative activity. Although our preliminary experiments showed that the primary REP cells in the mixed culture for 3 days expressed the *EpoGFP* gene in response to the PHDi, the cells lost their Epo-production ability within 1 week. Thus, it is proposed that the renal microenvironment, including tubulointerstitial and/or vasculointerstitial interactions, keeps REP cells quiescent in healthy kidneys and that damage to tubules and/or vessels in injured kidneys or in culture dishes induces the transformation of REP cells through activating proliferation and inactivating Epo production. Additionally, quiescence may be required for the Epo-producing ability of REP cells.

Replic cells preferentially grow and maintain their sharp configuration in a special culture medium optimized for mesenchymal stem cell (MSC) growth rather than in a general medium. In addition, Replic cells and REP cells commonly express CD73 at high levels^12,24,25^; CD73 is an MSC marker^47^. Thus, it is reasonable to conclude that REP cells include MSCs. Indeed, there are reports demonstrating that MSCs play essential roles in kidney development by secreting growth factors^48^, that renal interstitial fibroblasts in healthy kidneys and renal myofibroblasts in injured kidneys are derivatives of MSCs^5^, and that cells bearing MSC properties exist in kidneys for tissue repair^25,49,50^. Importantly, a subset of MSCs isolated from adult mouse kidneys expresses *Epo*^25^.

A variety of cell types have been reported as origins of myofibroblasts emerging in kidney diseases. For instance, interstitial fibroblasts such as REP cells^5–7^, bone marrow-derived cells^8,9^, tubular epithelial cells^3^ and vascular endothelial cells^4^. The myofibroblastic features of Replic cells clearly demonstrate that REP cells are one of the sources of renal myofibroblasts. This conclusion coincides with our previous articles reporting that the majority of renal myofibroblasts in injured mouse kidneys originate from REP cells^6,7^.

TGFβ is known as a master regulator of fibrosis^2,51^, but its source is controversial in renal fibrosis. Injured tubular epithelial cells, endothelial cells, and macrophages are major TGFβ-producing cell candidates in fibrotic kidneys^52–54^. On the basis of data from Replic cells, we further argue that cell-autonomous TGFβ signalling promotes the myofibroblastic transformation of REP cells. We and others previously demonstrated that activation of TGFβ signalling, which is defined by the phosphorylation of Smad2/3, is detected in MF-REP cells in injured kidneys^7,30^. However, further studies using Replic cells are required to identify triggers of cell-autonomous TGFβ induction in myofibroblasts of injured kidneys. In addition to TGFβ, MCP1 and CXCL5 are secreted at high levels from Replic cells, and both chemokines have been suspected to be secreted from injured kidneys and to contribute to inflammation and fibrosis by recruiting myeloid cells from the bone marrow^55,56^. Analyses of Replic cells will allow us to deeply understand the molecular mechanism of organ fibrosis.

Hypoxia-inducible Epo production in REP cells is regulated at the gene transcription level^23^. In Replic cells exhibiting features of MF-REP cells, the promoter region of the *Epo* gene is highly methylated. Because DNA methylation in gene promoters blocks the association of basic transcription factors and RNA polymerases^57^, it is proposed that REP cells lose their Epo-production ability due to *Epo* gene silencing by DNA methylation during the transformation to myofibroblasts in injured kidneys. Similarly, a previous report demonstrated that deficient Epo production is caused by hyper-methylation of the *Epo* gene promoter in renal myofibroblasts^30^ although this study could not exclude the possibility that H-RAS overexpression but not myofibroblastic transformation directly mediated the DNA methylation. This content is confirmed by our experiment showing that the Epo-production ability of Replic cells was not induced by overexpression of HIF2α, a major activator of *Epo* gene transcription in REP cells^15^. In this study, we revealed that the expression of HIF2α at the mRNA and protein levels is blunted due to hyper-methylation of the *Epas1* promoter region in Replic cells. Therefore, epigenetic silencing in the *Epo* and *Epas1* loci is involved in the development of renal anaemia.

We previously reported that the Epo-production ability is retained beyond myofibroblastic transformation by the constitutive activation of HIF2α in REP cells before injury^15^. During Replic cell establishment under normal air conditions, HIF2α was probably inactivated, and the *Epo* and *Epas1* promoter regions were methylated. We then propose that active HIF2α or active *Epo* gene transcription may protect the *Epo* gene promoter from hyper-methylation in renal myofibroblasts. Overexpression of the constitutively active H-RAS may also contribute to the DNA-methylation-mediated gene silencing, because RAS signalling has been reported to induce DNA methylation primarily through the MAPK pathway^58,59^. Although the molecular mechanism of gene-specific methylation in renal myofibroblasts has not been elucidated, TGFβ signalling is supposed to induce DNA methylation through enhancing the expression of DNA methyltransferases in renal myofibroblasts^15,30^. This study proposes that *de novo* methylation of specific genomic regions, including the *Epo* and *Epas1* gene promoters, is constitutively induced by cell-autonomous TGFβ production in renal myofibroblasts.

Using mouse models, we previously demonstrated that MF-REP cells restore REP cell properties by ameliorating kidney injury^7^. Although this study failed to recover the Epo-production ability in Replic cells by TGFβ signal blockage, the inhibition of TGFβ signalling partially debilitated the myofibroblastic phenotype of Replic cells in the gene expression profile. Further attempts to restore REP-cell properties in Replic cells are directly linked to the identification of therapeutic targets for both renal fibrosis and anaemia in CKD. To treat renal anaemia, PHDi is effective for inducing renal Epo production in CKD patients, including end-stage renal disease patients^20^. However, the *Epo* gene in Replic cells is not responsive to PHDi. Our preliminary data show that the Epo-induction effect of PHDi is gradually weakened with the progression of kidney disease in mice. These observations suggest that REP cells may transform into myofibroblasts in a stepwise manner during kidney disease progression and that Replic cells may correspond to severely myofibroblast-transformed REP cells, in which Epo and HIF2α expressions are epigenetically silenced. Finally, we conclude that Replic cells will provide significant information on the molecular pathology of CKD, from which more than 10% of people suffer worldwide and for which no plausible treatments have been established due to a poor understanding of the mechanisms of CKD pathogenesis.

## Methods

### Ethics statement

All animal experiments were conducted in accordance with “Regulations for Animal Experiments and Related Activities at Tohoku University” by “Institutional Animal Care and Use Committee of Tohoku University (http://www.clar.med.tohoku.ac.jp/en.html; Approval No. is 2017-MdA-090)”.

### Mice

All animals, including ISAM-REC mice^13,14^, were maintained under specific pathogen-free conditions. Male mice at 11–14 weeks of age were used in all experiments. For UUO surgery, the left ureter was cut between 2 ligatures made by 4–0 Polysorb (Syneture) at the level of the lower pole of the kidney^7^. At 10 days after UUO surgery, both kidneys and plasma from the buccal vein were collected. Kidneys were fixed with 10% formalin (Nakalai Tesque) for 24 hours, and sections (5-µm thickness) of paraffin-embedded kidneys were stained with Masson’s trichrome stain (Polysciences). Kidney samples for RT-qPCR analyses were obtained 14 days after UUO surgery.

### Isolation and immortalization of REP cells

Kidneys of ISAM-REC mice were chopped and digested with 1.5 mg mL^−1^ of collagenase type II (Worthington) for 60 minutes at 37 °C followed by dissociation using a gentleMACS dissociator (Miltenyi Biotec). The cell suspension was incubated for one week, and adherent cells were infected with a lentivirus vector expressing the G12V oncogenic mutant of human H-RAS (abm). From the immortalized cells, REP-cell-derived cells positive for tdTomato expression were isolated using a FACS Jazz cell sorter with FACS Diva software (Becton Dickinson). In the isolated cell culture, a single colony was developed and expanded as the Replic cell line.

### Cell culture

MEFs were established from embryos of wild-type C57BL/6 mice (CREA Japan) at embryonic day 13.5. Replic cells, MEFs, and Hep3B cells (ATCC) were maintained in DMEM (Nakalai Tesque) containing 10% foetal bovine serum (FBS, Biosera) or MSCM (PromoCell) in a humidified atmosphere containing 5% CO_2_ at 37 °C. Unless otherwise noted, cells were seeded onto 6- or 12-well polystyrene culture plates (Merck) within 10 passages and harvested when the cells grew to 80–90% confluency. Cell images were taken using a BZ9000 fluorescence microscope (Keyence). The growth ratio of cells was examined daily using a Tali image-based cytometer (Thermo Fisher Scientific) after 1.0 × 10^4^ cells were seeded onto 3.5-cm dishes. For exposure to hypoxic air (consisting of 1% O_2_, 5% CO_2_, and 94% N_2_), cells were incubated in a Sci-tive hypoxia workstation (Ruskinn) for 24 hours.

### Transient transfection

A pCMX-HIF2αCA plasmid expressing mouse HIF2α, of which 2 specific prolyl residues were replaced with alanine residues to become resistant to PHD-mediated hydroxylation and constitutively active in mammalian cells^34^, and pEGFP-N1 (TaKaRa) expressing enhanced GFP were used for transient transfection assays. Cells with 80% confluency in a well of 6-well plates were transfected with a total of 600 ng of pCMX-HIF2αCA and/or pEGFP-N1 using Lipofectamine 2000 reagent (Thermo Fisher Scientific) and harvested 48 hours after transfection. Transfection efficiency was estimated by measuring the ratio of GFP-expressing cells in total cells using Tali.

### Compounds

SB431542 (Adooq Bioscience), GSK360A (Toronto Research Chemicals), and 5-Aza (Sigma-Aldrich) were resolved in dimethyl sulfoxide (DMSO, Nacalai Tesque) at 10 mM, 50 mM, and 5 mM concentrations, respectively. TGFβ N-Ab (1D11; Novus Biologicals) was dissolved in culture medium at a 2 mg mL^−1^ concentration just before administration. The resolved compounds were administered to the cells at a 1:1000 dilution. DMSO or culture medium was used as the vehicle control.

### Genomic PCR

Genomic DNA was purified with phenol/chloroform/isoamyl alcohol (Nakalai Tesque) from cells or mouse organs. Genotype PCR was performed with primers listed in Supplementary Table 1 using Ex-Taq polymerase (TaKaRa). Amplified DNA fragments were electrophoresed on 2% agarose gels containing ethidium bromide (Nakalai Tesque) and detected using an E-BOX imaging system with ultra-violet light (VILVER).

### Reverse transcription-quantitative PCR (RT-qPCR)

Total RNA was extracted and purified using ISOGEN (Nippon-Gene) immediately after cells were removed from incubators. For control samples, total RNA was purified from whole mouse kidneys using a Precellys 24-bead homogenizer (Bertin) and ISOGEN (Nippon-Gene). cDNA was synthesized using the reverse transcriptase from a Superscript III kit (Thermo Fisher Scientific). Quantitative PCR was performed with primers and probes listed in Supplementary Table 1 using FastStart SYBR green master mix (Roche) or TaqMan master mix (Roche) with a LightCycler 96 system (Roche). The expression levels of housekeeping *Hprt* (hypoxanthine phosphoribosyl transferase) mRNA were used as internal standards.

### Immunoblotting

Whole cell lysates were prepared using lysis buffer (50 mM Tris-Cl, 150 mM NaCl, 0.5% sodium deoxycholate, 0.1% SDS, and 1.0% NP-40) supplemented with MG132 (LifeSensors), Protease Inhibitor Cocktail (Nacalai Tesque), and Phosphatase Inhibitor Cocktail (Nacalai Tesque). The lysates were transferred to nitrocellulose membranes (Bio-Rad) after sodium dodecyl sulphate polyacrylamide (8.0%) gel electrophoresis (SDS-PAGE). The membranes were immunoblotted with primary antibodies against HIF1α (PAB12138; Abnova), HIF2α (C150132; LSBio), p-Smad2/3 (8828 S; Cell Signaling Technology), total Smad2/3 (8685 S; Cell Signaling Technology), or βTubulin (PA1-21153; Thermo Fisher Scientific). Subsequently, horseradish peroxidase (HRP)-conjugated secondary antibodies against rabbit (P0448; Dako or ab6802; Abcam), goat (205-035-108; Jackson ImmunoResearch Laboratories), or mouse (01803-44; Nacalai Tesque) immunoglobulins were used with chemiluminescence staining reagents (GE Healthcare) followed by detection and quantification of the signals using a C-DiGit blot scanner with an Image Studio software (LI-COR).

### Flow cytometry

Cells in culture plates were dissociated with accutase (Nakalai Tesque). Single-cell suspensions were stained with biotin-conjugated CD73 antibody (Thermo Fisher Scientific) or biotin-conjugated TGFβR2 antibody (BAF532; R&D Systems) for 30 minutes on ice and then washed and stained with streptavidin-APC for 30 minutes. After washing, flow cytometry was performed using FACS Jazz.

### Bisulfite sequencing

Genomic DNA was purified from Replic cells and chopped mouse organs incubated with proteinase K (Nakalai Tesque) overnight, and bisulfite conversion of genomic DNA was performed using an EpiTect Bisulfite Kit (Qiagen). Bisulfite-converted DNA of the *Epo* and *Epas1* gene promoters was amplified by PCR using primers listed in Supplementary Table 1. Subsequently, TA cloning of the amplified genomic DNA fragments into the pT7Blue T-vector (Novagen) was conducted. Eight clones for each promoter were bilaterally sequenced by using primers (Supplementary Table 1) annealing to the cloning site of the pT7Blue T-vector.

### Cytokine array

Subconfluent Replic cells and MEFs in 3.5-cm dishes were incubated with 2 mL of serum-free DMEM for 24 hours, and 1 mL of the culture supernatants were incubated with the membranes (Mouse Neuro Antibody Array III, Abcam), on which capture antibodies against 23 cytokines (including TGFβ) were printed as shown in Supplementary Fig. 3a. After a 2-hour incubation, the membranes were washed and then incubated with a cocktail of biotin-conjugated detector antibodies against each cytokine for 2 hours. Chemiluminescent signals were detected and quantified using C-DiGit after incubation of the membranes with HRP-conjugated streptavidin for 2 hours. All procedures were performed at room temperature.

### ELISA

Subconfluent Replic cells (4.43 ± 0.25 [×10^4^ cells]) or MEFs (4.00 ± 0.38 [×10^4^ cells]) in a well of 12-well plates were incubated with 1 mL of serum-free DMEM for 24 hours. Total TGFβ concentrations in these samples were measured using a Total TGFβ1 Pre-coated ELISA kit (BioLegend) according to the manufacturer’s instructions. Briefly, samples were appropriately diluted and incubated in a 96-well plate coated with anti-TGFβ1 capture antibody. After a 2-hour incubation, the cells were washed and then incubated with biotin-conjugated TGFβ detection antibody for 1 hour. Developed colour intensity was measured using a FilterMax F5 microplate reader (Molecular Devices) after incubation of the cells with HRP-conjugated streptavidin and fluorescent substrate.

### Statistics

The data are shown as the mean ± standard error (SE). Comparisons between groups were conducted using one-way ANOVA with the Tukey-Kramer test or two-tailed, unpaired Student’s *t*-test or χ^2^ test as appropriate. The data were considered statistically significant at *P* < 0.05.

## Supplementary information

Supplementary Information

## Data Availability

All data supporting the results of this study are available in the article and supplementary information files or are available from the authors upon request.
