# Supplementary material for: An immortalized cell line derived from renal erythropoietin-producing (REP) cells demonstrates their potential to transform into myofibroblasts
Source: Sci Rep. 2019 Aug 2;9:11254. doi: 10.1038/s41598-019-47766-5 (PMC6677766; doi:10.1038/s41598-019-47766-5)
Supplement: Supplementary file 1 — Supplementary Information [file 41598_2019_47766_MOESM1_ESM.docx]

**Supplementary information**

**An immortalized cell line derived from renal erythropoietin-producing (REP) cells demonstrates their potential to transform into myofibroblasts**

Koji Sato^1,2^, Ikuo Hirano^1,3^, Hiroki Sekine^1,4^, Kenichiro Miyauchi^1,2^, Taku Nakai^1^, Koichiro Kato^1^, Sadayoshi Ito^2^, Masayuki Yamamoto^5^ & Norio Suzuki^1^

**Author affiliations:**

^1^Division of Oxygen Biology, Tohoku University Graduate School of Medicine

^2^Division of Nephrology, Endocrinology, and Vascular Medicine, Tohoku University Graduate School of Medicine

^3^Department of Molecular Hematology, Tohoku University Graduate School of Medicine

^4^Department of Gene Expression Regulation, Institute of Development, Aging and Cancer, Tohoku University

^5^Tohoku Medical Megabank Organization, Tohoku University

***Correspondence:**

Norio Suzuki (sunorio@med.tohoku.ac.jp)

Division of Oxygen Biology, Tohoku University Graduate School of Medicine

2-1 Seiryo-Machi, Aoba-ku, Sendai, Miyagi 980-8575, Japan.

Phone: +81-22-717-8206; Fax: +81-22-717-8090

**Running headline**: REP-cell-derived cell line

Supplementary information: 6 figures and 1 table


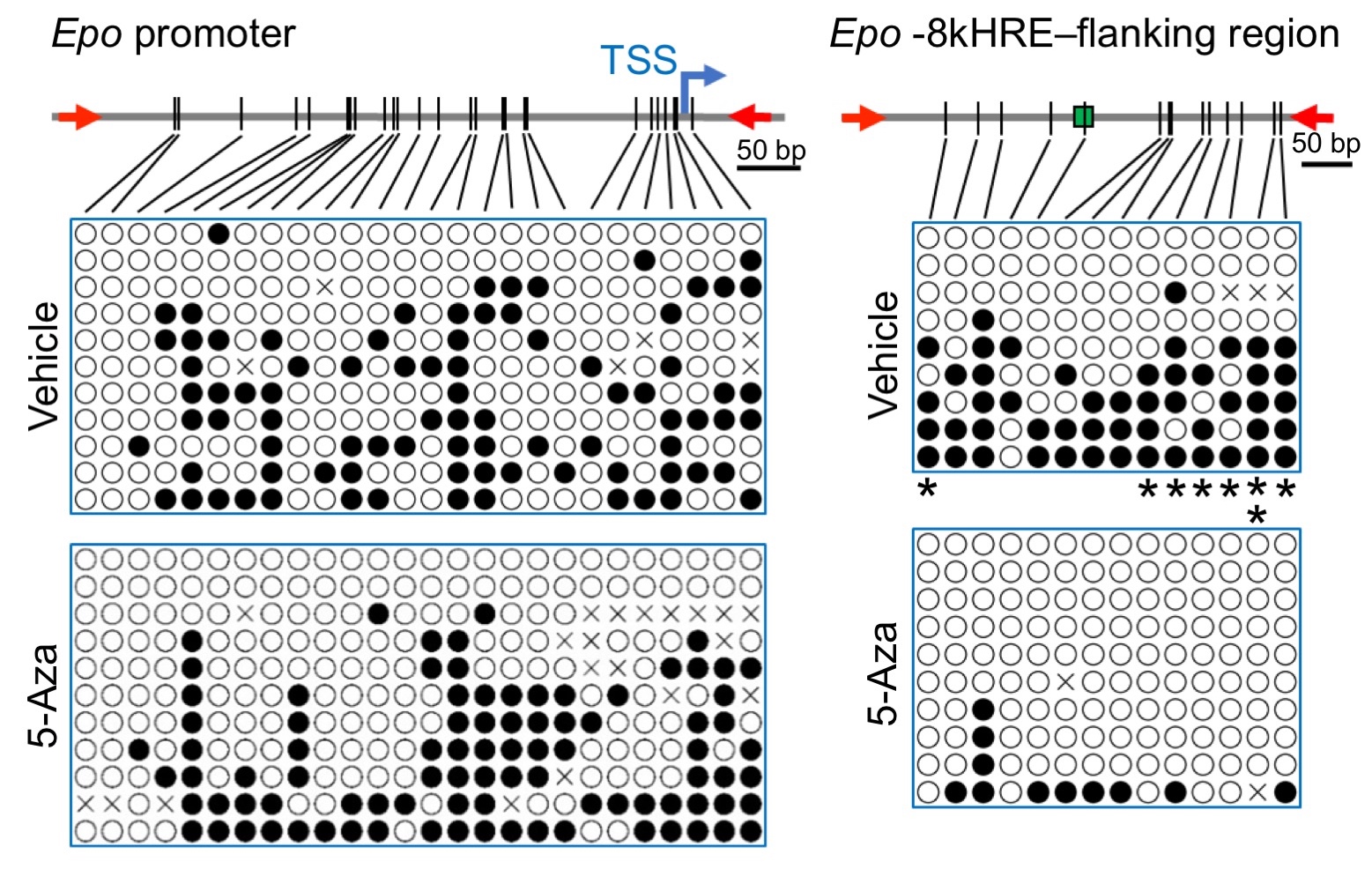


**Supplementary Figure 1**. **DNA methylation resistant to a DNMT1 inhibitor (5-Aza) in the *Epo* gene promoter region.** Summary of results from bisulfite sequencing in the promoter (left) and -8kHRE-flanking (right; localized 8-kb upstream from the transcription start site [TSS] of the mouse *Epo* gene)^32^ regions of the *Epo* gene in Replic cells cultured with (lower) or without (upper) 5 µM of 5-Aza for 7 days. Each row represents a single clone. Vertical bars indicate CpG sites in the tested regions (between red arrows), and white and black dots represent unmethylated and methylated CpG sites, respectively. “X” represents CpG sites that could not be read in the sequences. The green box indicates a putative HIF-binding sequence. *p < 0.05 and **p < 0.01 between the methylated ratio of each CpG site, using χ^2^ tests. Note that the promoter region was not responsive to 5-Aza treatment while the -8kHRE region was significantly demethylated by the treatment. Primer sequences used for amplification of the sequencing regions are 5’-ACC CTA AAA ATC ACA AAT CCT TAAC and 5’-TTT TTG GTT TAT TTT TTT GGA AAT TAG for the promoter region; 5’-AAT TGT TGG GTT GTG TTT TTA TGTT and 5’-CAA AAA ATC CCC AAA AAC TAA TCT AC for the -8kHRE-flanking region.


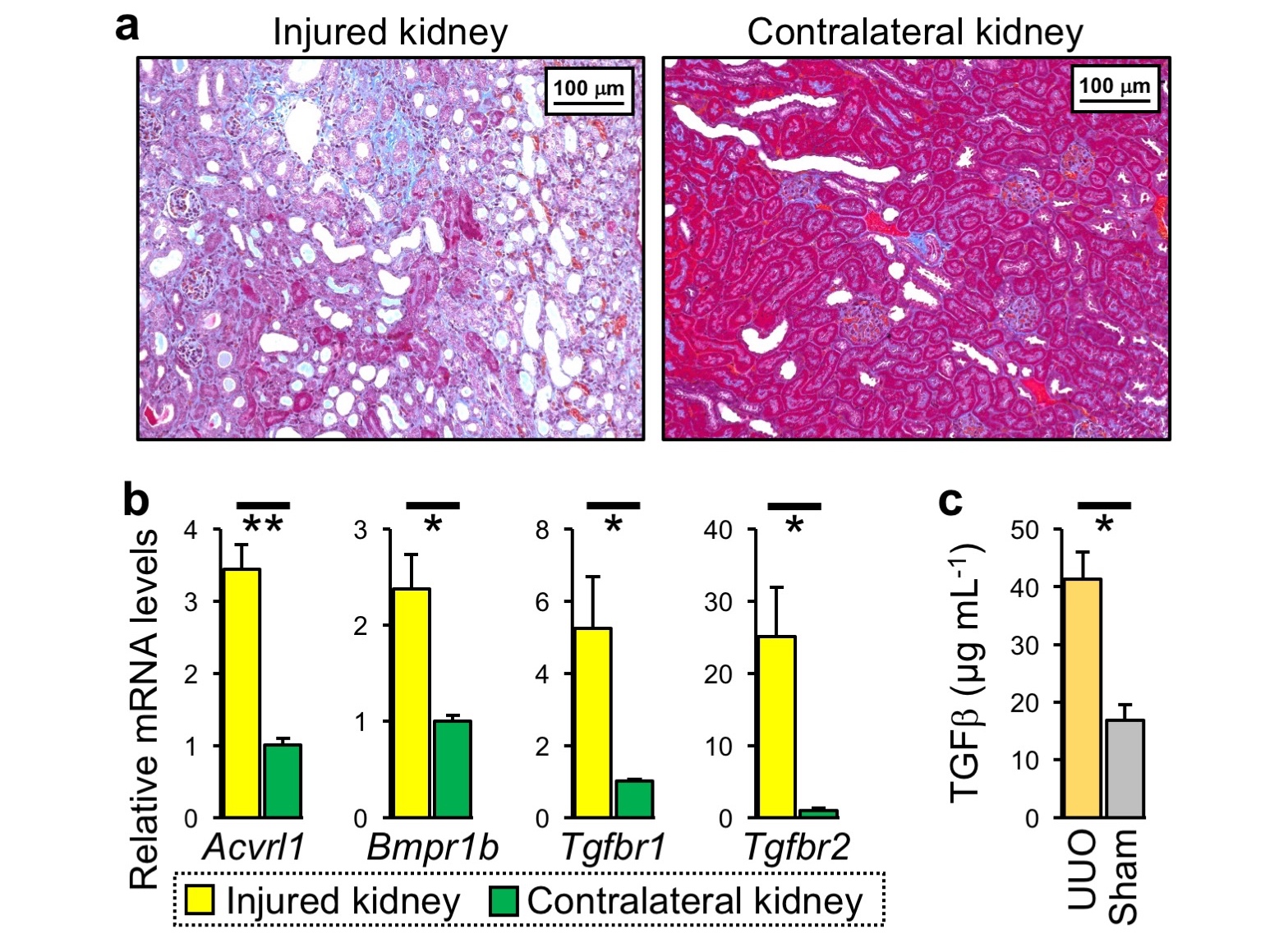


**Supplementary Figure 2. Kidney fibrosis induced by ureteral obstruction.** (**a**) Masson’s trichrome staining of kidney sections from a representative mouse subjected to UUO for 10 days. In the injured kidney (left), fibrotic areas (blue), damaged tubules (thin purple), and tubular lumens (empty) are expanded compared to the contralateral kidney (right), which is filled with healthy tubules (dark purple). (**b**) mRNA expression levels of genes for TGFβ superfamily receptors in the injured and contralateral kidneys of mice subjected to UUO for 14 days. The average expression level in contralateral kidneys was set at 1.0. n = 3 for each kidney. (**c**) TGFβ concentrations in plasma of UUO-subjected and sham-operated mice. n = 3 for each group. Error bars are standard errors. *p < 0.05 and **p < 0.01 by two-tailed, unpaired Student’s *t*-tests.


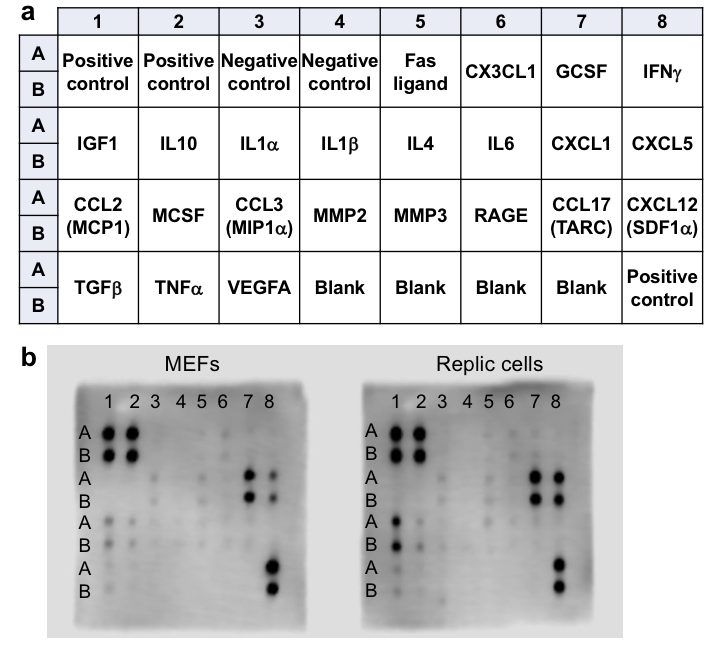
**Supplementary Figure 3. Antibody array of culture supernatant of MEFs and Replic cells.** (**a**) Mapping of antibodies on membranes on Mouse Neuro Antibody Array III (Abcam). Each antibody against the indicated factors has duplicate spots (A and B). (**b**) Scanned images of the antibody array data of supernatants from MEFs (left) and Replic cells (right). The chemiluminescent intensity of each spot was quantified in Figure 5e.


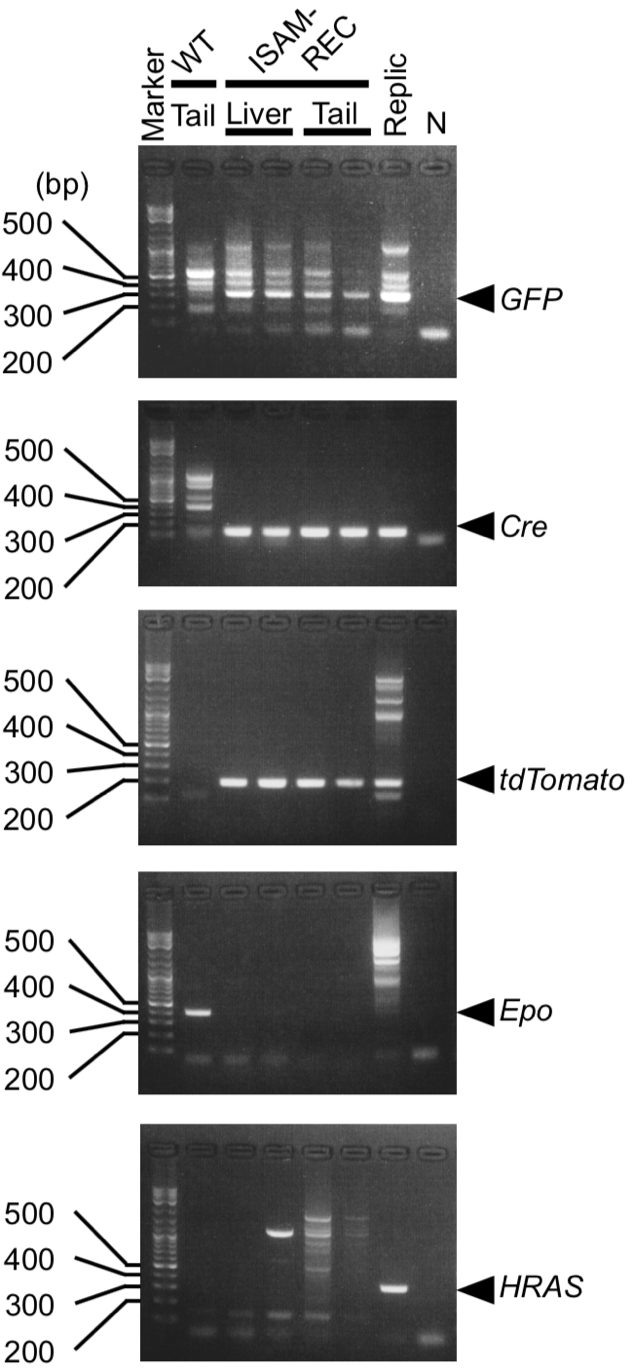


**Supplementary Figure 4. Full scan images of data shown in Figure 1e.**


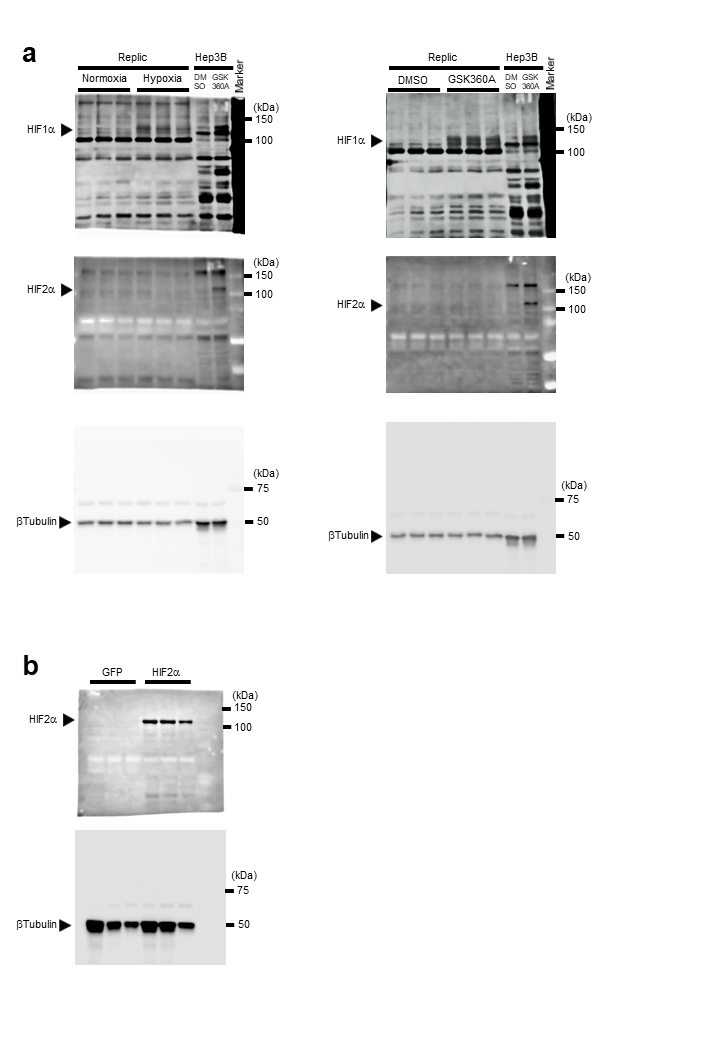
**Supplementary Figure 5. Full scan images of data shown in Figure 3c (a) and 4b (b).**

**
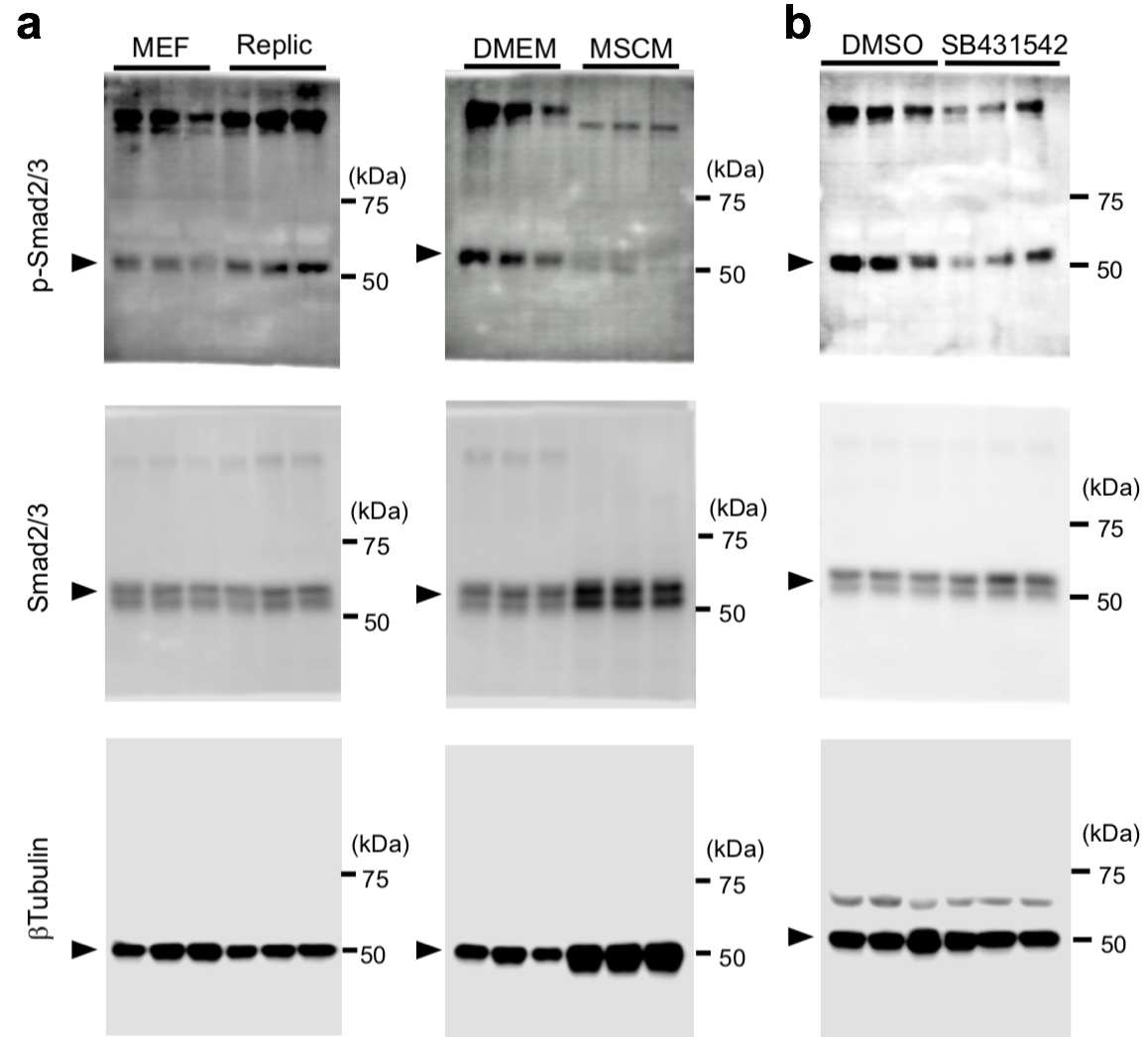
**

**Supplementary Figure 6. Full scan images of data shown in Figure 6a (a) and 7c (b).**

**Supplementary Table 1. Oligo-nucleotide sequences used in this study.**

| **Target** | **Primer 1 (5’ – 3’)** | | **Primer 2 (5’ – 3’)** |
| --- | --- | --- | --- |
| **For genotyping PCR** | | | |
| *GFP* | CTGAAGTTCATCTGCACCACC | GAAGTTGTACTCCAGCTTGTGC | |
| *Cre* | ACGTTCACCGGCATCAACGT | CTGCATTACCGGTCGATGCA | |
| *tdTomato* | CTGTTCCTGTACGGCATGG | GGCATTAAAGCAGCGTATCC | |
| *Epo* | ACAGGAAGGTCTCACATAGCC | TGGGGAAACCCCCATGAGATC | |
| *HRAS* | AGGCATCCTCCACTCCCTG | AGCAGGTGGTCATTGATGGG | |
| **For RT-qPCR** | | | |
| *Hprt* | CTGGTGAAAAGGACCTCTCG | TGAAGTACTCATTATAGTCAAGGG | |
|  | (FAM-labelled probe: ATCCAACAAAGTCTGGCCTGTATCCAAC) | | |
| *Nt5e* | CGCTCAGAAAGTTCGAGGTGTG | CGCAGGCACTTCTTTGGAAGGT | |
| *Pdgfrb* | CGGCCTGTGACTAGAAGTCC | GAGCTTGAGGCGTCTTGG | |
| *Kdr* | CGAGACCATTGAAGTGACTTGCC | TTCCTCACCCTGCGGATAGTCA | |
| *Cdh1* | GGTCATCAGTGTGCTCACCTCT | GCTGTTGTGCTCAAGCCTTCAC | |
| *Nphs1* | GCATAGCCAGAGGTGGAAATCC | GAACGGTCATCACCAGCACACT | |
| *EpoGFP* | GGTGGATCCTAAAGCAGCAG | GAAGACTTGCAGCGTGGAC | |
| *Vegfa* | CAGGCTGCTGTAACGATGAA | CTATGTGCTGGCTTTGGTGA | |
| *Slc2a1* | CCATGGATCCCAGCAGCAAG | CCAGTGTTATAGCCGAACTGC | |
| *Serpine1* | AGGATCGAGGTAAACGAGAGC | GCGGGCTGAGATGACAAA | |
| *Hif1a* | CCTGCACTGAATCAAGAGGTTGC | CCATCAGAAGGACTTGCTGGCT | |
| *Epas1* | GGACAGCAAGACTTTCCTGAGC | GGTAGAACTCATAGGCAGAGCG | |
| *Acta2* | CCCACCCAGAGTGGAGAA | ACATAGCTGGAGCAGCGTCT | |
| *Fn1* | ACCGACAGTGGTGTGGTCTA | CACCATAAGTCTGGGTCACG | |
| *Tgfb1* | TGGAGCAACATGTGGAACTC | CAGCAGCCGGTTACCAAG | |
| *Acvrl1* | GACATGACTTCGCGGAACTC | ACTCTTGAGGTCACGATGGG | |
| *Bmpr1b* | GCGCTATATGCCTCCAGAAG | CTCCTTGCAATCTCCCAGAG | |
| *Tgfbr1* | TCGACGCTGTTCTATTGGTG | CAACCGATGGATCAGAAGGT | |
| *Tgfbr2* | ATCTGGAAAACGTGGAGTCG | TCACTTCTCCCACAGCATTG | |
| **For bisulfite sequencing** | | | |
| *Epo* | GTGTGTGTTTGGAATAGTTTGTTTT | TCTATAAAACCAAATCACCCCACTA | |
| *Epas1* | GAAGGAGGAGGAAGGGTAGG | AACCATACAATCTCAAAACACTACC | |
| pT7Blue-T | CAGGCTTTACACTTTATGCTTCC | CGATTTCGGCCTATTGGTTA | |
